# Brazilian version of the Critical Care Functional Rehabilitation Outcome Measure: translation, cross-cultural adaptation and evaluation of clinimetric properties

**DOI:** 10.5935/0103-507X.20220025-en

**Published:** 2022

**Authors:** Luiz Alberto Forgiarini Júnior, Brenda Lilja da Fontoura, Desirée Ribeiro Kobylinski, Soraia Genebra Ibrahim Forgiarini, Vinicius Maldaner

**Affiliations:** 1 Postgraduate Program in Health and Human Development, Universidade La Salle - Canoas (RS), Brazil.; 2 Course of Physiotherapy, Centro Universitário Metodista IPA - Porto Alegre (RS), Brazil.; 3 Faculdades Integradas de Taquara - Taquara (RS), Brasil.; 4 Program of Health Sciences, Escola Superior de Ciências da Saúde - Brasília (DF), Brazil.

**Keywords:** Physical therapy modalities, Exercise therapy, Mobility limitation, Recovery of function, Critical illness, Outcome assessment, Health care, Inpatients, Surveys and questionnaires, Intensive care units

## Abstract

**Objective:**

To translate, crossculturally adapt and evaluate the clinimetric properties of the Critical Care Functional Rehabilitation Outcome Measure for evaluating the functionality of patients admitted to intensive care units in Brazil.

**Methods:**

The process of translation and cross-cultural adaptation involved the following steps: initial translation, synthesis, back-translation, expert committee review and pretesting. The intra- and interrater reliability and agreement were analyzed between two physical therapists who evaluated the same group of patients (n = 35). The evaluations were performed by each therapist independently and blinded to the score assigned by the other professional. The qualitative analysis was performed by the review committee, and the experts adapted and synthesized the Portuguese translation of the Critical Care Functional Rehabilitation Outcome Measure.

**Results:**

There was agreement between the initial Brazilian translations of the Critical Care Functional Rehabilitation Outcome Measure scale. The conceptual, idiomatic, semantic and experimental equivalences between the original and translated versions were assessed, resulting in the final Brazilian version of the scale, called the *Medida de Resultado da Reabilitação Funcional em Cuidados Intensivos*. The evaluation of the clinimetric properties showed evidence of a high degree of agreement and reliability, as all had an intraclass correlation coefficient above 0.75. The overall intraclass correlation coefficient was 0.89.

**Conclusion:**

The translated version of the Critical Care Functional Rehabilitation Outcome Measure scale for assessing the functionality of patients admitted to an intensive care unit can be used reliably in Brazil following translation and cross-cultural adaptation to Brazilian Portuguese and presents evidence of excellent interrater reliability.

## INTRODUCTION

Neuromuscular complications are common in critically ill patients and can be severe and persistent, with long-term repercussions on both the functionality and quality of life of patients. Muscle weakness acquired in the intensive care unit (ICU) is multifactorial, with direct causes, such as systemic inflammation, and indirect causes, such as time on mechanical ventilation, that contribute to complications related to immobility. Thus, the early rehabilitation of these individuals plays an important role in reducing the deleterious effects associated with ICU stay and in improving the functionality of these patients at the time of hospital discharge.^(1)^

These neuromuscular changes are evaluated by physiotherapists who are directly involved in the rehabilitation process. The presence of these professionals in ICUs results in a lower degree of respiratory complications, neuromuscular improvement, greater physical function, better quality of life, and a reduction in the lengths of hospital stay and mechanical ventilation.^(2)^ Thus, tools that can help these professionals identify functional changes in individuals hospitalized in these units are of great value for the early identification of physiological changes and possible therapeutic strategies.

Existing validated scales for evaluating the functionality of patients admitted to the ICU include the Physical Function in Intensive Care Test score (PFIT-s), Chelsea Critical Care Physical Assessment Tool (CPAx), Perme Intensive Care Unit Mobility Score, Surgical Intensive Care Unit Optimal Mobilization Score (SOMS), Intensive Care Unit Mobility Scale (IMS) and Functional Status Score for the Intensive Care Unit (FSS-ICU).^(3)^ However, a simple instrument for multidisciplinary teams to assess critical patients quickly, easily and objectively remains lacking.^(4)^

Among the instruments developed to evaluate the functionality of patients admitted to the ICU, the Critical Care Functional Rehabilitation Outcome Measure (CcFROM) scale, developed and validated by Twose et al.^(5)^, is a fast, simple and low-cost instrument that can help diagnose and guide physiotherapeutic care. The scale consists of nine motor tasks, graded from zero to seven, for a total score ranging from zero to 63, where 63 represents complete independence; however, only an English version is available, which makes it difficult to use by Brazilian professionals.

The translation, cross-cultural adaptation and evaluation of the clinimetric properties of the scale into the Portuguese language would allow professionals who deal with critically ill patients to have at their disposal a simple functional assessment instrument adapted specifically to critical Brazilian patients. Furthermore, this is the only scale developed to measure outcomes related to the rehabilitation of critically ill patients and can be used initially in the intensive care unit but can potentially be used later in the general ward environment.

The term “clinimetry” was introduced by Alvan R. Feinstein in the early 1980s to describe a domain related to evaluation indices and scales. It has a set of rules that govern the structure of the indices and the choice of variables present in the instrument, and the evaluation of consistency and validity.^(6)^ The relevant clinimetric properties to be considered when selecting an instrument include the ability to measure what is intended (validity) and the ability to obtain accurate results within or between evaluators (intra- and interevaluator reliability, respectively).^(3)^ The clinimetric properties of the original CcFROM instrument have already been published and have shown good results.^(5)^

Based on this information, the objective of the present study was to translate, cross-culturally adapt and evaluate the clinimetric properties of the CcFROM scale to assess the functionality of patients admitted to the ICU in Brazil.

## METHODS

This is a cross-sectional study that followed the recommendations of the current guidelines.^(7)^ The execution of the project was authorized by Dr. Paul W. Twose, author of the original instrument. This study was submitted to and approved by the Research Ethics Committee of the *Complexo Hospitalar Santa Casa de Misericórdia de Porto Alegre* (opinion No. 2,628,187).

### The Critical Care Functional Rehabilitation Outcome Measure scale

The CcFROM scale was developed and validated by Twose et al. to assess the functionality of patients admitted to the ICU with key functionality items. The nine functional tasks of the CcFROM are elevation of the extended leg, rolling, moving from lying to sitting, sitting balance, moving from sitting to standing, standing, stationary gait, transfer from bed to chair and walking. Each item is graded on an eight-point scale, with zero for incapacitated or not tested; one for total assistance (four or more therapists); two for maximum care (three therapists); three for moderate care (two therapists); four for minimal assistance (one therapist); five for supervision (maximum of one therapist); six for modified independence (increased time to perform the activity alone) and seven for complete independence. The total score ranges from zero to 63, and 63 represents complete independence.^(4)^

### Translation and cross-cultural adaptation

#### Stage I - Initial translation

The translation of the original version of the scale was performed by two independent professionals who were competent in the English language and whose native language was Portuguese (Brazil). Translator 1 (T1) was a health care professional with experience in assessing the functionality of patients admitted to the ICU and was aware of the concepts that were examined in the translated scale. Translator 2 (T2) was a health professional but did not have knowledge about the concepts being examined.

#### Stage II - Synthesis

The versions translated by T1 and T2 were compared and analyzed to produce a single text (T1.2). A consensus approach was used to resolve differences via a meeting between the researcher responsible for the study and the translators of Stage I. Then, the consensus-based translation of the instrument was developed (T1.2).

#### Stage III - Back-translation

In this step, the consensus version (T1.2) was backtranslated into English by two independent translators, producing back-translation 1 (BT1) and back-translation 2 (BT2). The translators were both native English speakers and competent in Portuguese. Of these, one was unaware of the original version and was not in the health field (teacher trained in language study), and the other was employed in the health field and had knowledge of the concepts addressed in the scale.

#### Stage IV - Review by an expert committee

A panel of experts was formed, the Translation Panel, consisting of four health professionals (three of whom participated in Stages I and II) and an individual with a bachelor of arts and linguistics. This committee of experts received the five versions (T1, T2, T1.2, BT1 and BT2) and discussed each item of the instrument to resolve differences in translations and other discrepancies. The Translation Panel tried to make the best possible use of the linguistic specialization of its members, solving the following types of disagreements: conceptual (referring to differences in the conceptual formulation of the evaluation), idiomatic (different linguistic expressions), semantic (differences related to the content of the test) and experiential (cultural differences). After this step, the final version of the scale was generated.

Reproducibility describes the similarity of results obtained through repeated measures in a clinically stable sample. In this study, the reproducibility of the Brazilian version of CcFROM was evaluated by two qualified physical therapists who received standardized training on the CcFROM scale. The therapists evaluated the scale independently and blinded to the evaluation of the other professional. “Reproducibility” is an umbrella term for two properties called reliability (relative error of the measurement) and agreement (absolute error of the measurement).

#### Stage V - Pretest

A pretest was performed to verify whether the version was equivalent to the original scale and whether the target group to be evaluated would understand the scale instructions well.

The objective of this phase was to correct possible semantic errors, concepts and interpretations of the scale.

For the pretest, the six physical therapists of the intensive care unit of a public hospital in Porto Alegre (RS), each with at least 1 year of experience in the unit, were recruited. The physiotherapists read the scale, explained their responses and reported any type of problem with the instrument. No physical therapist reported difficulties in interpreting and understanding the questions.

### Clinical evaluation of the scale

The selected patients were admitted to the Central ICU of the *Hospital Santa Clara* of the *Complexo Hospitalar Santa Casa de Misericórdia de Porto Alegre*. All patients or their legal guardians agreed to participate and signed informed consent forms. The inclusion criteria were either sex, age greater than 18 years and use of mechanical ventilation for more than 72 hours during their ICU stay. Patients with cognitive impairment, traumatic-orthopedic or rheumatologic pathology and who did not agree to participate in the study were excluded.

### Data analysis

The qualitative analysis was performed by the review committee, in which the experts adapted and synthesized the translation of the CcFROM scale into Portuguese. The normality of the data was assessed using the Shapiro-Wilk test and the Kolmogorov-Smirnov test. The data from the quantitative analysis are expressed as the mean and standard deviation or absolute and percentage values. The intraclass correlation coefficient (ICC), using the absolute agreement method, was calculated to evaluate the reliability between the two evaluators. An ICC above 0.75 indicates good to excellent reliability.^(7)^ The internal consistency was analyzed using the Cohen test. The interrater agreement was analyzed using the Bland-Altman test. A histogram demonstrating the floor and ceiling effects was generated with JMP software 16 (SAS Institute Inc., Cary, NC).

## RESULTS

The cross-cultural adaptation of the CcFROM scale to the Portuguese language produced two versions, the T1 and T2 translations. The discrepancies and issues addressed were discussed by the committee to produce a single text (T1.2). When the translations were different, the most common terms were used. For some items, changes were made by the translators to improve the idiomatic and semantic equivalence between the items of the originallanguage scale and the Brazilian version of the CcFROM.

The main terms discussed were “raise”, “Stand”, “Marching on Spot” and “Walking”. Table 1S of the supplementary material shows the summary of this process.

The back-translation into English again showed differences with the original version; for example, “Straight leg raise” in the original version was translated back into English as “Raising extended leg”. Four items in the synthesis version of the translations and six items in the synthesis version of the back-translations were considered slightly altered compared with the original version. All other items are shown in table 2S, and the final version of the scale is shown in table 3S (Supplementary material).

A total of 35 patients were included in the study between June and August 2018. There was a predominance of female patients. The mean age of the patients was 59 ± 16 years, and respiratory disorders predominated (Table 1). The patients spent an average of 18 days in the ICU and 12 days on mechanical ventilation.

**Table 1:** Characteristics of the sample evaluated during the pretest stage with the *Medida de Resultado da Reabilitação Funcional em Cuidados Intensivos* scale, Brazilian version

Variable	
Age (years)	59 ± 16
Sex, female	20 (57)
APACHE II	18 [17 - 21]
Diagnosis at ICU admission	
Respiratory	11 (31.4)
Gastrointestinal	11 (31.4)
Sepsis	3 (8.5)
Cardiovascular	4 (11.7)
Trauma	3 (8.5)
Neurological	3 (8.5)
Time on MV (days)	9 [5 - 16]
Length of Hospitalization (days)	16 [9 - 11]

APACHE II - *Acute Physiology and Chronic Health Evaluation II*; ICU - intensive care unit; MV - mechanical ventilation. Data are expressed as the mean ± standard deviation, n (%) or median [interquartile range].

During the pretest phase, the physical therapists did not report any uncertainty or problems with the interpretation that affected their performance; thus, no additional modifications were made to the Brazilian Portuguese version.

There was very good reliability between the two observers for all tasks and for the total CcFROM score. ICC values above 0.75 indicated good to excellent reliability; the reliability ICC was 0.81 for the “lying to sitting” item, which was nevertheless considered to indicate good reliability (Table 2).

**Table 2:** Values of the intraclass correlation coefficients for the tasks of the *Medida de Resultado da Reabilitação Funcional em Cuidados Intensivos* scale

Task	Intraclass Correlation Coefficient (95% CI)
Raising extended leg	0.89 (0.81 - 0.94)
Rolling	0.91 (0.87 - 0.95)
Laying to sitting	0.81 (0.73 - 0.86)
Sitting balance (sitting on the edge of the bed for at least 10 seconds)	0.92 (0.87 - 0.94)
Sitting to standing	0.90 (0.83 - 0.94)
Standing (for at least 10 seconds)	0.87 (0.83 - 0.92)
Marching in place (at least 10 steps)	0.91 (0.84 - 0.93)
Moving from bed to armchair	0.90 (0.87 - 0.93)
Walking (minimum of 10 steps)	0.88 (0.84 - 0.92)
Total scale score	0.89 (0.85 - 0.93)

95%CI - 95% confidence interval.

**Figure 1:**
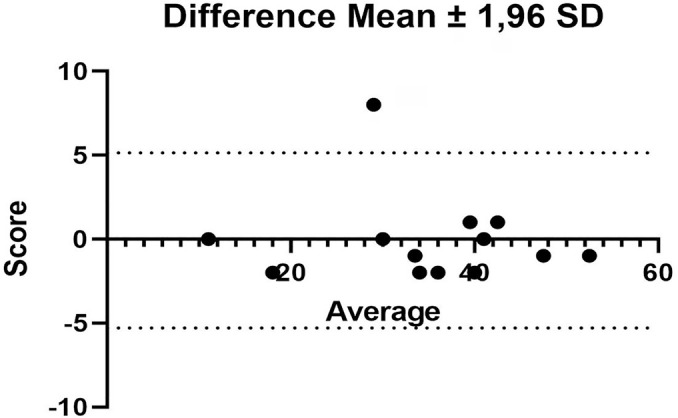
Analysis of agreement between evaluators of the *Medida de Resultado da Reabilitação Funcional em Cuidados Intensivos*. Mean difference - 0.07 with standard deviation 2.66 using the Bland-Altman test. SD - standard deviation.

**Figure 2:**
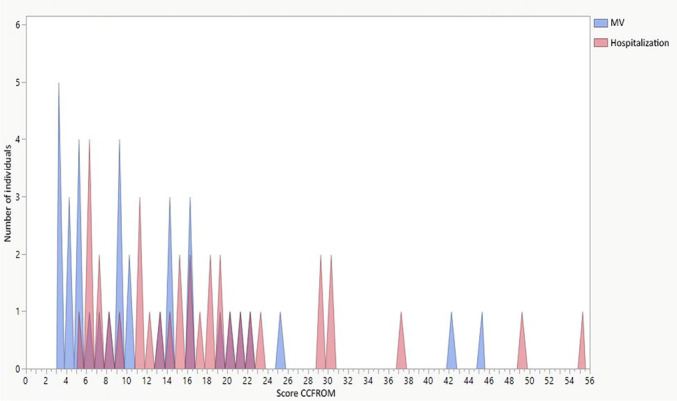
Histogram of the distribution of patient scores from the *Medida de Resultado da Reabilitação Funcional em Cuidados Intensivos* scale. MV - mechanical ventilation.

The internal consistency was good to excellent according to the Cohen test. Cronbach’s alpha ranged from 0.90-0.94. The interrater agreement was analyzed using the Bland-Altman test, and the mean difference was -0.07, with a standard deviation of 2.66 (Figure 1). The analysis of the floor effect (minimum score) and ceiling effect (maximum score) showed a minimum ceiling effect (2%) for the application of the scale at ICU discharge. No patient reached a score of zero upon discharge from the ICU, and thus there was no floor effect (Figure 2).

After translation and validation, the name Critical Care Functional Rehabilitation Outcome Measure was changed to *Medida de Resultado da Reabilitação Funcional em Cuidados Intensivos* for the final version.

## DISCUSSION

This was the first study to perform an official translation and cross-cultural adaptation to the Brazilian Portuguese language and clinimetric evaluation of the CcFROM scale.

Translation alone of the CcFROM instrument would not have been sufficient for application in Brazil; adaptation to Brazilian culture was necessary.^(8)^ The process of cultural adaptation is designed to achieve equivalence in relation to language (semantic and idiomatic equivalence) between the original and translated versions. Cross-cultural adaptation is a procedure that must be self-performed for different countries, cultures and languages and requires a rigorous methodology, as these processes are as important as the construction of a new instrument.^(8^.^9)^ Adapting an existing instrument is more efficient and less costly than the development of a new tool.^(9)^

The CcFROM scale is more relevant at ICU discharge than upon admission to the unit, as it evaluates physical functions that require greater patient ability.^(3)^ These data reinforce the importance of the translation and cross-cultural adaptation of the instrument. Other instruments for assessing functionality and mobility have also been developed, translated and adapted for the specific evaluation of patients in the ICU.^(4)^ The Perme scale, developed for measuring the mobility status and standardize the evaluation of patients in the ICU, has a score ranging from zero to 32 points and is divided into 15 items grouped into seven categories: mental state, potential barriers to mobility, strength functional, bed mobility, transfers, assistive devices for ambulation and resistance measurements. A high score indicates high mobility and the need for less assistance, while a low score demonstrates low mobility and a greater need for assistance,^(10)^ which is similar to the CcFROM scale score.

Unlike the Perme scale, the PFIT-s scale is a physical function test that involves four components: assisted sitting, stationary gait, and muscle strength of the shoulder flexors and knee extensors.^(11)^ This instrument can be used to guide the prescription of exercises within the ICU, as well as to measure functional recovery.^(11^.^12)^

The FSS-ICU scale, already validated in Brazil, was evaluated on 30 patients. This measure aims to evaluate the physical function of patients admitted to the ICU, similar to the CcFROM, but it has five tasks (rolling, moving from the supine to sitting position, moving from the sitting to the standing position, sitting bedside and walking). The CcFROM, on the other hand, has nine tasks: the five of the FSS-ICU, and four additional tasks: elevation of the extended leg, standing (at least 10 seconds), transfer from bed to chair and stationary gait (at least 10 steps). Both have an ordinal scale of eight points ranging from zero (totally unable to perform) to seven (complete independence).^(13)^

The results of the present study show evidence of a high degree of agreement and reliability according to the analyses performed with the Brazilian version of the CcFROM scale. It was found that the CcFROM showed excellent interrater agreement and reliability for most of the domains evaluated.

The high ICC value (0.89) found for interrater reliability demonstrates the consistency of the measure. In addition, the instrument proved to be homogeneous and adequately internally consistent, since ICC values above 0.75 are considered high and reliable.^(7)^

After development, the original instrument was subjected to evaluation of its clinimetric properties, showing strong results in terms of interrater and interrater reliability, with ICCs of 0.985 and 0.901, respectively.^(4)^ The results from the analysis of the original instrument corroborate those of the Brazilian version of the CcFROM, as the values for interrater reliability were similar to those of the analyses performed in Brazil.

The Perme scale and the IMS were validated in Brazil and evaluated in 103 patients, most of whom were male. This differs from the CcFROM, which was evaluated in a predominantly female population. The mean age of the patients evaluated by the scales were similar: 52 years for the Perme and IMS and 56 years for the CcFROM. Regarding the reason for hospitalization, respiratory disorders predominated in both studies.^(14)^

The Perme scale and the IMS showed a high degree of agreement and reliability, with an ICC of 0.99.^(14)^ Silva et al.,^(13)^ after evaluating 30 patients, demonstrated that the Brazilian version of the FSS-ICU had good reliability between the evaluators for the scores of each of the five tasks and for the overall scores (intraclass correlation coefficient ranged from 0.88 to 0.91).

Analyzing these studies, we can observe that the degree of agreement and intra- and interrater reliability of the CcFROM was good to excellent, with interclass correlation coefficients between the scores of the two raters ranging from 0.81 to 0.92.

Even after cross-culturally adapting the instrument, which strictly followed the methodology proposed for this type of study, it was not guaranteed that the instrument would maintain the clinimetric properties of the original instrument. This can be explained by the cultural differences between the associated populations, because the people of the world do not share a common culture and lifestyle.^(9)^

Verification of the clinimetric properties refers to the study of the properties of the tools and instruments of clinical evaluation according to the assessments performed in the present study. The need to include clinimetric evaluations for instruments that evaluate functionality made this type of verification emerge in an extremely relevant way for clinical investigation and patient care. The term “clinimetry” refers to a domain related to evaluation indices and scales. This is because clinimetry has a set of rules that govern the structure of the indices, the choice of variables present in the instrument and the evaluation of consistency and validity. In addition, it provides information for clinical judgment, which directly influences the results of treatment, both in research and in clinical practice.^(5,15)^

A good instrument for assessing functionality should have a relevant scoring scale, clinical utility, reliability, evidence of adequate validity, practicality, ease of application and interpretation and low cost,^(6,16,17)^ all of which were demonstrated by the CcFROM.

Thus, the Brazilian Portuguese version of the CcFROM provides Brazilian physiotherapists with an important assessment tool for use in clinical practice and research given its psychometric power, applicability and external validity.

The CcFROM should be used in the evaluation of physical function in the ICU environment and does not require any additional equipment. It can also be easily integrated into the usual clinical care provided by the physical therapist. There are several pieces of evidence that report the consequences of the ICU admission process and its risk factors, including a decline in functionality with a negative impact on the quality of life of individuals in both the short and long term,^(18-21)^ and a tool that assists in the evaluation of these critically ill patients would be of great value.

It is expected that this study will be of benefit to professionals working in the care area and to researchers and professors by providing a valid and reliable instrument for identifying the functional limitations of patients admitted to Brazilian ICUs; for directing and monitoring patient physical therapy plans; and for evaluating the treatment results and the impact of ICU stay on functionality.

## CONCLUSION

The Brazilian version of the Critical Care Functional Rehabilitation Outcome Measure, called the *Medida de Resultado da Reabilitação Funcional em Cuidados Intensivos* can be used reliably in Brazil to evaluate the functionality of patients admitted to the intensive care unit because it has been translated and cross-culturally adapted into Brazilian Portuguese and presents evidence of excellent reliability among evaluators.
