# Supplementary material for: Brazilian version of the Critical Care Functional Rehabilitation Outcome Measure: translation, cross-cultural adaptation and evaluation of clinimetric properties
Source: Rev Bras Ter Intensiva. 2022 Apr-Jun;34(2):272–8. doi: 10.5935/0103-507X.20220025-en (PMC9354108; doi:10.5935/0103-507X.20220025-en)
Supplement: Supplementary file 1 [file rbti-34-02-0272-suppl01.pdf]

Brazilian version of the Critical Care Functional Rehabilitation Outcome Measure: translation, cross-cultural adaptation and evaluation of clinimetric properties

Versão brasileira da Critical Care Functional Rehabilitation Outcome Measure: tradução, adaptação transcultural e avaliação das propriedades clinimétricas

Luiz Alberto Forgiarini Júnior[1], Brenda Lilja da Fontoura[2], Desirée Ribeiro Kobylinski[2], Soraia Genebra Ibrahim Forgiarini[3], Vinicius Maldaner[4]

Table 1S - Translations of Translator 1 and Translator 2 and final Brazilian version of the Critical Care Functional Rehabilitation Outcome Measure scale

Original item	T1 and T2	T1.2
Straight leg raise	T1 - *Elevação da perna reta*<br>T2 - *Elevar a perna estendida*	*Elevar a perna estendida*
Rolling<br>(left or right)	T1 - *Rolar (para o lado direito ou esquerdo)*<br>T2 - *Rolar (esquerda ou direita)*	*Rolar*<br>*(para o lado direito ou esquerdo)*
Lie to sit	T1 - *Deitado para sentado*<br>T2 - *Deitado para sentado*	*Deitado para sentado*
Sitting balance<br>(sit on edge of bed for a minimum 10 seconds)	T1 - *Equilíbrio sentado (sentar à beira do leito por no mínimo 10 segundos)*<br>T2 - *Equilíbrio sentado (sentar beira leito por no mínimo 10 segundos)*	*Equilíbrio sentado*<br>*(sentar à beira do leito por no mínimo 10 segundos)*
Sit to stand	T1 - *Sentado para em pé*<br>T2 - *Sentado para em pé*	*Sentado para em pé*
Stand<br>(minimum 10 seconds)	T1 - *Ficar de pé (por no mínimo 10 segundos)*<br>T2 - *Em pé (pelo menos 10 segundos)*	*Ficar de pé*<br>*(por no mínimo 10 segundos)*
Marching on spot<br>(minimum 10 steps)	T1 - *Marcha estacionária (no mínimo 10 passos)*<br>T2 - *Marcha no lugar (mínimo 10 passos)*	*Marcha estacionária*<br>*(no mínimo 10 passos)*
Transfer bed to chair	T1 - *Transferência da cama para a cadeira*<br>T2 - *Transferência do leito para poltrona*	*Transferência da cama para a cadeira.*
Walking<br>(minimum 10 steps)	T1 - *Deambular (mínimo de 10 passos)*<br>T2 - *Andar (mínimo de 10 passos)*	*Deambular*<br>*(mínimo de 10 passos)*

T1 - Translator 1; T2 - Translator 2; T1.2 - consensus based on the translations of Translators 1 and 2.

Table 2S - Back-translation by Back-translator 1 and Back-translator 2 and final version of the back translation of the Critical Care Functional Rehabilitation Outcome Measure scale from the Brazilian version

Original version	BT1 and BT2	BT1.2
Straight leg raise	BT1 - Raising extended leg<br>BT2 - Raising extended leg	Raising extended leg
Rolling<br>(left or right)	BT1 - Rolling (left or right)<br>BT2 - Rolling (left or right)	Rolling<br>(left or right)
Lie to sit	BT1 - Laying to sitting<br>BT2 - Laying to sitting	Laying to sitting
Sitting balance<br>(sit on edge of bed for minimum 10 seconds)	BT1 - Seated balance (sitting at bedside for at least 10 seconds)<br>BT2 - Sitting balance (sit on edge of bed for minimum 10 seconds)	Sitting balance<br>(sit on edge of bed for minimum 10 seconds)
Sit to stand	BT1 - Sitting to standing<br>BT2 - Sitting to standing	Sitting to standing
Stand<br>(minimum 10 seconds)	BT1 - Standing (at least 10 seconds)<br>BT2 - Standing (at least 10 seconds)	Standing<br>(at least 10 seconds)
Marching on spot<br>(minimum 10 steps)	BT1 - Marching in place (at least 10 steps)<br>BT2 - Marching in place (at least 10 steps)	Marching in place<br>(at least 10 steps)
Transfer bed to chair	BT1 - Moving from bed to armchair<br>BT2 - Moving from bed to armchair	Moving from bed to armchair
Walking<br>(minimum 10 steps)	BT1 - Walking (at least 10 steps)<br>BT2 - Walking (at least 10 steps)	Walking<br>(at least 10 steps)

BT1 - Back-translator 1; BT2 - Back-translator 2; BT1.2 - consensus based on the translations of Back-translators 1 and 2.

Table 3S - Scores of the *Medida de Resultado da Reabilitação Funcional em Cuidados Intensivos* scale

Score	
7	Complete independence
6	Modified independence (for example, needing extra time)
5	Supervision (maximum 1 therapist)
4	Minimum assistance (assistance from 1 therapist)
3	Moderate care (assistance from 2 therapists)
2	Maximum assistance (assistance from 3 therapists)
1	Total assistance (assistance from 4 or more therapists)
0	Incapable/not tested (includes the use of mechanical aids, for example, use of a sling or hoist to stand)
Total (maximum score = 63)	
